# Imagining a lean and agile digital health ecosystem – a measure of pandemic responsiveness

**DOI:** 10.7189/jogh.10.020391

**Published:** 2020-12

**Authors:** Zainab Samad, Sana Mahmood, Sameen Siddiqui, Zulfiqar A Bhutta

**Affiliations:** 1 Aga Khan University, Karachi, Pakistan; 2 SickKids Centre for Global Child Health, Toronto, Canada

The COVID19 pandemic has laid bare stark inadequacies and disparities in health systems all over the world, particularly so in low/middle income countries [1]. Recent events highlight that strong political will and prior preparedness for health-related emergencies are critical to being able to think and act at the same time. A necessary requirement is rapid access to, and synthesis of, the right information. In a time of crisis, the question we are all asking ourselves is, *how do we come together, leverage our strengths and resources to combat this enormous challenge?* The other question we need to be asking ourselves is, how can our *systems* be set up to do the same? A digital health system that is smart, agile, performs early warning functions and is programmed to detect and inform on inequities in health care, can be a life-saving tool, especially in low resource settings.

In such environments, strategies and their implementation modalities must be grounded in local contextual information on available resources and needs. Although traditional methods of data gathering and strategy delivery are often labor intensive, and slow, “data trails” of a digitally connected population offer unique opportunities for effective health surveillance, emergency preparedness and response capacity.

Pakistan, for example, struggles with a scarce skilled research and health professional workforce, but it has over 165 million registered mobile phone users (76% teledensity), 76 million 3G/4G subscribers [2]. This extensive digital coverage represents a relatively unexploited electronic platform for strengthening health surveillance. Indeed, back in 2012, real-time telephone triage based data were used to perform fine-grained forecasting of Dengue outbreaks. These predictions were shared with public health officials and became a critical component of government-led strategies to contain Dengue [3]. While this is an important example of the nimble, need-responsive use of digital resources for population health, more deliberate, cohesive, and sustainable approaches utilizing available data resources are required.

The Electronic Health Record (EHR) is another key component of a digital health system that becomes an especially helpful tool where large numbers of diversely skilled health care professionals involved in delivering health care do not exist. In Pakistan, few if any public sector apex health centers have EHR and digital records, which makes information tracking difficult. The implementation of EHR for such settings must be reimagined such that it is not a burdensome exercise of “documentation for billing purposes”. Instead it must be lean, collecting only information relevant to patient care and physician communication. While providing information for disease surveillance, EHR can also be used as a change effector at both local and population levels – as a physician aid through implementing simplified automated algorithms, managing simple screening and follow up, and for critical public health messaging through a necessary connection to more commonly used digital platforms such as cell phones.

**Figure:**
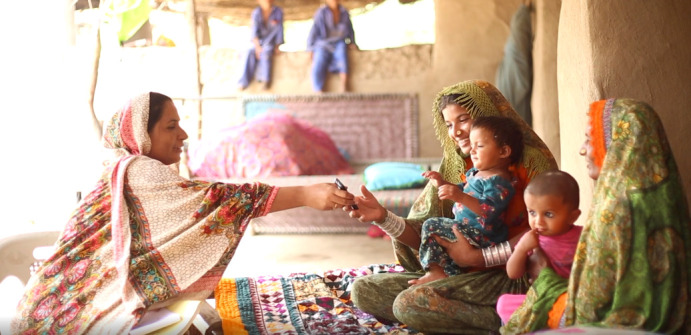
Photo: From the archives of the Aga Khan University.

For Pakistan, this means an integration and connection of the currently available digital health platforms which include District Health Information System (DHIS), Lady health worker Management information system (LHW – MIS) with EHR in settings where it has been implemented, electronic disease early warning systems (eDEWS) [4] and finally Union council maintained vital statistics. This critically needed digital integration rests on creating common data standards and interoperability of the various systems. Strategic data sharing between governmental agencies and academia and/or public and private sectors would allow crowd-sourcing of digitally literate individuals, research capacity and may help in establishment of an effective surveillance-implementation relationship. Additionally it is important to consider in this schema the systematic use of de-identified digital exhausts. The latter include geolocations offered by cell phone data, publicly available symptoms, behavior information gleaned from social media websites, all data that have been used successfully in forecasting outbreaks and providing insights into population health [5]. As we pull in these key variables we must ensure we consider equity and the social determinants of health in the design of these systems. This requires that health care workers collaborate with the information technology workforce *and* social scientists; a workforce facile in principles of research, data and health is created; that EHR systems connect with and serve the most vulnerable populations, such as those affected by poverty, gender or geography; that there is community engagement and buy-in, and that a digital system considers connectivity of all segments of a population; and that comprehensive data informs policy. While 76% of Pakistan’s population subscribes to telecom services, there is only 36% internet penetration and a wide gender and geographic digital divide. Even among phone users how many are women? Even if women are registered mobile phone users, is the device ‘managed’ by them or a male family member and will the data submitted provide a clear picture of those that most require health services? A laudable government effort, a WhatsApp ChatBot to provide information on Coronavirus, will likely reach the 82% male and 18% female users of the application [2,6]. Our data collection is only useful if it is designed to collect information in a comprehensive, culturally sensitive and inclusive manner ‘leaving no one behind’ – in the era of Sustainable Development Goals [7].

Connecting the data exhaust from ubiquitously used digital platforms such as cell phones, mobile health applications, geospatial mapping systems, to electronic health records to finally feed into a massive ‘smart’ disease surveillance platform is technically possible and the need of the hour. For example, an organized effort to gather data on patients being tested, positive cases and outcomes is being undertaken by the Government of Pakistan. Coalescing this data with virtual data exhausts, geospatial information or deep dives with input from academicians and health professionals to understand disparities by gender, income status and ethno-geography is the next step in building a large-scale disease surveillance platform, that is also equity focused. This type of country wide pandemic collaborative would be populated by multiple trusted data streams – both structured and unstructured – along with data analytics and visualizations components. These data streams would include but are not limited to prior survey information (both in the public/non-public domain), geospatial satellite imagery, nascent or established hospital information systems, district health information systems, web scrubbing from digital media, and government maintained COVID-19 data. Lessons from other countries reflect that the establishing of Event Based Surveillance (EBS) systems such as a country wide pandemic collaborative, is successful if there is integration with existing systems. EBS systems were found to be particularly useful in guiding outbreak response in countries where existing systems were not strong [8]. In Bangladesh for example, “routine health facility based, indicator-based surveillance in a set of hospitals was complemented by EBS in a way that clusters of meningo-encephalitis cases were notified immediately and investigated to identify small area Nipah outbreaks” [8]. Pakistan is primed for this type of integration with numerous independent systems that need to be brought together for an agile and equity sensitive response.

It behooves each nation, whether high or low income, to have a handle on its own unique disease patterns, challenges, resources, and emerging threats. For LMICs there is a real opportunity that a digital health system can serve as a cost-effective platform through which decision-making considers all segments of a population. Such a digital health system is not only a requirement for evidence informed allocation of resources, but necessary for internal and external accountability of health systems and their financiers.
